# [Retracted] Sinomenine hydrochloride inhibits cell survival in human hepatoma Huh7 cells 

**DOI:** 10.3892/br.2025.1984

**Published:** 2025-04-28

**Authors:** Ying Wang, Ming Li, Xuesong Yu, Ali Chen, Ying Ding, Yan Wang, Yan Wang

Biomed Rep 8:510-516, 2018; DOI: 10.3892/br.2018.1084

Following the publication of the above paper, it was drawn to the Editor’s attention by a concerned reader that certain of the flow cytometric data shown in Fig. 2 on p. 513 were strikingly similar to data that had appeared previously in other papers written by different authors at different research institutes.

In view of the fact that the abovementioned data had already apparently been published prior to its submission to *Biomedical Reports*, the Editor has decided that this paper should be retracted from the Journal. The authors were asked for an explanation to account for these concerns, but the Editorial Office did not receive a reply. The Editor apologizes to the readership for any inconvenience caused.

